# Progress of the Medical Sciences

**Published:** 1914-09

**Authors:** 


### Progress of the Medical Sciences.

### MEDICINE.

**Diet in Diseases of the Heart, Vessels and Kidneys.**—Dr. Karl Frenkel[1] gives a useful summary of recent work on this subject. With regard to the *heart*, the object of dietetic treatment is to strengthen and maintain its muscular capacity. So long as there is likely to be active endocarditis the patient should be placed on fever diet, with restriction of fluids.

In chronic heart disease the aim is to bring the patient into a normal state of nutrition, and to keep him there. Superfluous fat makes the work of the heart difficult, and leads to its early failure. Defective nutrition is equally injurious. Eppinger has shown that in this condition an hypertrophied gives way before a sound heart. In obese cardiac patients dietary restriction should not be carried beyond a certain point, and further removal of fat should be gradually accomplished by carefully regulated exercises. Only if the patient is unable to get about should dietary restrictions for the removal of fat be actively pushed.

Oertel advised a diet rich in proteids, up to 150 grammes per diem, but later authors limit the amount from 80 to 120 grammes. All authors advise either a very limited use of alcohol, or total abstinence, especially from strong alcoholic drinks, *e.g.* brandy and liqueurs. Beer is inadvisable, not so much on account of its alcoholic content, but because of the amount of fluid, and of the sugar it contains, which provides superfluous nutrient material. Strauss advises coffee, especially prepared to diminish the amount of caffein. Weak tea, coffee and soups may be taken in large amounts, unless contra-indications exist. The use of tobacco must be strictly limited.

With regard to the amount of fluid, in well-compensated heart disease, most writers restrict the quantity; some insist on fluid being taken only at meal times, whilst others make no

[1] Schmidt's *Jahrbücher*, 1914, cccxix. 124.

restriction. The balance of evidence seems in favour of a moderate restriction of fluid.

In failure of compensation meals should be small, and intervals of feeding more frequent. In view of the failure of the digestive juices, and of the loss of appetite, the food must be varied in character, in order that the patient may be induced to take enough to maintain his nutrition and that of the heart-muscle, which is the all-important point. In acute heart failure the temporary stimulating effect of alcohol should be utilised. In the milder forms of disturbance of compensation, such as dyspnœa and nocturnal micturition, in addition to rest in bed and the administration of cardiac tonics, the amount of fluid is to be limited to $1\frac{1}{4}$ or $1\frac{1}{2}$ litres daily. This restriction of fluid increases the efficacy of digitalis. Spices and salt should be forbidden, the elimination of the latter by the kidneys is difficult, and in the most severe cases of heart disease may fail as it does in parenchymatous nephritis, whilst retention of salt leads to retention of water in the tissues. Whilst in mild disturbance of compensation restriction of fluid seems to promote recovery, V. Noorden, Kraus and Strauss consider that it does not do so in severe cases, and they remove all restriction if the patient suffers much from thirst, or is losing his appetite.

For severe cases of cardiac dropsy, in which digitalis has failed, the milk cure, first employed by Karell and since modified by Lenhartz and Jacob, has come into general use. For five to seven days the patient takes 300 cc. milk, hot or cold, four times daily, at four hourly intervals. Nothing else is taken, he stays in bed during the whole cure. Generally a pronounced diuresis sets in on the third or fourth day. The condition of the blood pressure varies. During the next two to six days, between the feeds of milk, an egg, rusks, some black bread, vegetable soup and meat is allowed. On the thirteenth day he returns to his ordinary diet, with the exception that for two to four weeks the total daily quantity of fluid is limited to 800 cc. If the first cure is not successful, a repetition of it after two to three weeks, with the administration of cardiac tonics, often brings about the desired result. Causes of failure of the cure are that the heart disease is far advanced, or is complicated by nephritis or uræmia. The success of this treatment is attributable to the rest given to the organs of digestion and assimilation by the temporary restriction of nutriment and fluid, and to the absence from the food of salt and of irritative substances.

With regard to *arterio-sclerosis*, there is nothing fresh to report as to dietetic treatment. Most authors advise the limitation of proteid foods to an amount not exceeding 60 to 80 grammes daily, with the use of milky foods, vegetables and nuclein-free foods. When symptoms appear many recommend an exclusively milk diet for two weeks.

Opinion is practically unanimous as to the advisability of restriction of salt in all forms of *nephritis*, especially in those forms in which there is œdema, or any tendency to it. As regards food, this is attained by (1) baking bread without salt ; (2) using butter made without salt ; (3) limiting the quantity of milk to 1 litre or less daily ; (4) all cooking to be done without salt ; (5) soups and broths are forbidden ; (6) vegetables to be washed a long time in water before cooking.

In cases of contracted granular kidney without uræmic symptoms, the daily amount of proteid should be reduced to 80 grammes. 80 to 110 grammes is considered necessary by v. Noorden, and every week on one day only 20 to 30 grammes is allowed. The latter amount would be contained in a dietary composed of bread, cereal foods, fruit, vegetables, butter, and half a litre of milk. Less proteid than the above is insufficient to maintain the nutrition of the cardiac muscles, one of the most important considerations in chronic renal disease. In order to dilute the uræmic poisons, as well as to wash out the tissues, Strauss endeavours to excite free diuresis. For this purpose he gives in contracted kidney with good cardiac compensation, and in the first stage of chronic uræmia, enough fluid to produce a daily flow of 1500 to 2000 cc. of urine. If at the onset of uræmia so much fluid cannot be taken by the mouth, he orders, even in the presence of œdema, drop-instillation of pure water per rectum. If heart weakness threatens the amount of fluid drunk is reduced by half to three-quarters litre, or the Karell milk cure (see above) is instituted. If heart weakness and uræmic symptoms occur simultaneously, the former is treated by rest and digitalis, and the amount of fluid ingested is not diminished. Herz advocates a similar course of treatment. v. Noorden is against the use of such large amounts of water, because he thinks that it may lead to heart failure early in the case. He allows the patient sufficient water to keep the quantity of urine at 1300 to 1500 cc., because the researches of Mohr and Dapper show that nitrogenous excretion is not hindered until the daily quantity of urine falls below 1250 cc. Every week the patient is allowed to drink as much water as he pleases on one day, and if the heart condition is good, for a period of fourteen days in every two to three months a " drink cure " is carried out, the patient taking two to three litres of water daily. v. Noorden justifies the restriction of fluid by his observation that it brings about a fall of blood pressure from 30 to 50 mm. Hg. Other writers think the best results in cases of contracted kidney are given by following a middle course, and allow 1½ litres of water per diem.

In acute glomerular and parenchymatous nephritis, in the exacerbations of chronic parenchymatous nephritis and of chronic interstitial inflammations, the excretion of water by the kidney is disturbed, as a primary symptom, and careful consideration of this factor is needed. If too much fluid is given, the result is an increase of œdema. The amount of fluid should therefore be restricted, so far as the patient's thirst, which is often great, allows, to about 1500 cc., and elimination promoted by vapour baths, until a spontaneous diuresis is established. When diuresis is free all authors advise administration of abundant fluid.

Full references to the recent literature are given at the end of this article, *q.v.*

**On the present state of our knowledge of "Occult" Hemorrhages from the Stomach and Intestines.**—Prof. Boas discusses this question in an exhaustive paper.[1] In the first place it is necessary to exclude traces of blood coming from outside the stomach and intestines, *e.g.* from the nose, mouth, throat, lungs, etc. ; and secondly a still more important source of confusion arises from blood-containing foods, *e.g.* all kinds of meat, fish, which will give a reaction with the delicate tests employed indistinguishable from that due to small hemorrhages (exogenous occult blood). The more delicate the tests the more likely are mistakes from this latter source. The rule generally given is that for the guaiac-turpentine test four days' abstinence from flesh and fish is sufficient to exclude errors of this kind. Boas considers this period much too short for the recent modifications of the guaiac-turpentine and the more delicate tests now used ; moreover, the absence of muscle fibres from the fæces, on which reliance has been placed, does not exclude a positive reaction to the blood tests from exogenous (food) material.

Information as to the origin of occult blood from stomach or intestine can be obtained from simultaneous examination of the stomach contents and the fæces. If both give a positive reaction, the inference as to source is certain. If one is positive and the other negative, further observations over a period of a few days will as a rule make clear the source of the hemorrhage. In every case examination of both stomach contents and fæces for occult blood should be carried out. There are no distinguishing characteristics between the reactions given by small hemorrhages (endogenous blood) and those given by meat or fish diet (exogenous blood) With the tests in present use the patient should abstain from flesh and fish for seven to ten days ; vegetables containing chlorophyll should also be excluded. It is not necessary in every case for the preliminary fast from meat and fish to be so lengthy : with the use of fish and white

[1] Schmidt's *Jahrbücher*, 1914, cccxix. 225–237.

meats the guaiac-turpentine test generally soon becomes negative, and a strongly positive reaction after a few days points clearly to the endogenous origin of the blood.

For the distinction between simple ulcer and carcinoma, especially of the stomach, occult blood will very soon, generally within fourteen days, disappear in cases of ulcer ; in those of cancer it will continue in spite of dietetic and medicinal treatment.

Only seldom is a single testing sufficient to show occult blood, but in well-marked cases two to four observations suffice to establish the diagnosis. In most affections, especially in ulcer, the cessation of occult bleeding is of equal diagnostic importance to its presence. Only three tests of those in use need be considered :—

1. The original guaiac-turpentine test. Boas advises a modification of the original method. Instead of water the fæces are mixed with glacial acetic acid and alcohol (1 : 3) : this is a more powerful solvent for hæmatin than the acetic ether formerly used. A small portion of the fæces are rubbed up with 20 cc. of acetic alcohol solution in a porcelain dish, filtered through a moist filter, allowed to stand, and then filtered again. If the filtrate is a deep brown, from the alcohol taking up colouring matter of fæcal and vegetable origin, it is diluted with alcohol to a yellowish colour, and then guaiac-turpentine added in the ordinary way.

2. The benzidine test. This test is about eight times more delicate than the first one. It is unnecessary to describe the ordinary method of carrying it out, but when the amount of blood is extremely minute, the reaction may be interfered with through anticatalytic bodies in the fæces, which are especially abundant on a vegetarian diet. This inhibitory action may be prevented by boiling the small portion of fæcal matter used, or by triturating with glacial acetic acid. Boas for this test also recommends the use of acetic acid and alcohol.

3. The phenolphthalein test. The principle of this test lies in the fact that in the presence of blood the colourless phenolphthalin is changed into phenolphthalein. According to the ordinary mode of doing the test, it lies midway in delicacy between the first two tests. Boaz finds that by using the glacial acetic alcohol described above for extracting the fæces the reaction is much more delicate : it has the advantage of being sharper (better defined) than the others. The reagent is made by dissolving phenolphthalein one grm., potass. hyd. fus. 25 grm. in water 100 cc., and adding powdered zinc 10 grm. The mixture is heated over a small flame with constant shaking until completely decolourised, and is then filtered whilst hot.

To the filtered extract of fæces prepared as in the other tests ten to fifteen drops of the solution are added, and three to five drops $H_2O_2$ solution. In the presence of much blood the mixture is a carmine red, with smaller quantities a rose red, and in minimal amounts reddish. Excess of acetic acid is to be avoided, as it would render the reaction negative, and if the mixture gives an acid reaction a few drops of liq. potossæ should be added. The patient must, of course, not take aperients containing phenolphthalein, and must be eight to ten days on a diet free from flesh and fish, before it is certain that a positive reaction is due to endogenous blood.

In order to get the fullest information from these tests observations must be carried out daily, that the course of the occult bleeding may be followed. It is hardly necessary to add that all vessels and test tubes employed must be scrupulously clean. The investigation of occult blood aids in the diagnosis between peptic ulcer and cancer. The presence of blood at first in undoubted quantity, then gradually diminishing, and finally disappearing, is in favour of an ulcer, whilst the steady persistence of bleeding speaks strongly for a malignant growth.

There are not infrequent cases in which severe pains occur after meals, but in the absence of other positive signs the diagnosis between the neuroses and inflammatory lesions cannot be made ; in such cases a systematic investigation for occult blood will often clear up the case.

In cases of acute peptic ulcer an attack of hæmatemesis or melæna is followed by a longer or shorter post-hemorrhagic stage, in which only occult blood is found. The same condition is found in those cases of chronic ulcer, with a long history of hyperchlorhydria with pain, in which sooner or later a sudden hæmatemesis or melæna occurs. There are further cases of ulcer of very long duration, in which no manifest hemorrhage ever occurs. In these cases, with the delicate tests in present use, the presence of occult blood can almost always be demonstrated. So long as an ulcer, whether acute or chronic, is not healed there must be bleeding, though it may be in minimal quantity.

If on the other hand, in spite of clinical symptoms of gastric ulcer, blood is persistently absent, there can be no active ulceration, and the symptoms are due to some of the numerous complications, *e.g.* hyperacidity, perigastritis, pyloric stenosis, hour-glass stomach, etc.

A negative result is important in another direction as an aid in the often difficult distinction of ulcer from affections in which the stomach is secondarily disturbed, such as cholelithiasis, atypical cases of chronic appendicitis, and affections of the kidneys.

To sum up, in an active ulcer sufficiently careful investigation will always show the presence of occult blood, and where a diagnosis of ulcer has been made and no bleeding is found, the diagnosis is either incorrect or the symptoms are due to a healed ulcer with complications, for a healed ulcer without complications should give no trouble.

Further, is the presence of occult blood always due to an ulcer, or may it be due to hemorrhagic erosions ? The author thinks it impossible to be certain on this point, nor does he consider it important, for such cases should be treated as ulcer.

With regard to other kinds of ulceration, especially tuberculous, nothing certain is known as to occult blood. Statements as to its presence are at variance—there are some positive, but mostly negative findings—and the published material is still too small, and not sufficiently verified by post-mortem examinations, for any positive statement to be made.

**Occult blood in malignant growths of the alimentary canal.**— The clinical importance of occult bleeding in carcinoma rests upon the fact that the large majority of all carcinomatous growths tend to ulcerate sooner or later, irrespective of their position in the alimentary canal, and as this ulceration shows no tendency to heal, it follows that occult blood, once present, tends to persist permanently. The constant presence of occult blood with other clinical signs suggesting carcinoma is therefore a most important aid in diagnosis.

In carcinoma of the œsophagus, occult blood, though very frequently present, is not very important, because of the other means of diagnosis available in this situation.

In cancer of the stomach occult blood is present in the fæces in 95 per cent., the Mayo Brothers say 100 per cent. of cases, where the diagnosis has been confirmed by operation or post-mortem. Conditions which favour a negative reaction are great stenosis of the pylorus, obstinate constipation, and where the small hemorrhages are not thoroughly mixed with the fæces. But in such cases, if the patient is put on a fluid diet, and the actions rendered loose, a positive result will generally be obtained.

The discovery of occult blood is of great help in the differential diagnosis of—

1. Cases of gastric cancer, without a palpable tumour, and in which the only symptoms are those of profound anæmia.

2. Cases of scirrhus of pylorus, with presence of free HCl in gastric contents. Occult bleedings may decide the question of a benign or malignant stenosis of pylorus.

3. The development of a simple chronic into a malignant ulcer : the persistent presence of occult blood is in favour of the latter.

4. Similarly it points to a tumour in the upper abdomen of doubtful nature as one of gastric origin.

5. The test may be useful in ascites of uncertain origin.

In carcinoma of the colon Prof. Boas states that the test is positive in 85 per cent. of cases. A possible source of failure of the test is stenosis of the bowel, so that small quantities of blood fail to pass through, and in a single examination are missed. He recommends then that the bowel should be thoroughly washed out, and the last washings examined for blood. The importance of the test is practically confined to growths originating between the cæcum and splenic flexure: below this other means of diagnosis are now available.

Boas has no doubt of the value of occult bleedings in the early diagnosis of abdominal malignant disease, for they are often present much earlier than the ordinary pathognomonic signs of tumour, and we are therefore in a position to make a positive diagnosis in an earlier stage than was possible before their importance was recognised.

Lastly, the presence of occult blood is an aid to treatment, by indicating the nature of the food, etc., suitable to the case. Attention to this point will often save the patient on the one hand from being kept unnecessarily long on a fluid diet, and on the other from being allowed to get about and to take ordinary diet too soon. Careful investigation of the fæces for occult blood should be carried out over sufficiently long periods of time in all cases of gastric ulcer if the treatment is to be conducted on rational principles.

J. Michell Clarke.

### SURGERY.

**The Surgical Treatment of Gastric Ulcer.**—This subject has recently been carefully considered by Prof. Georg Perthes,[1] of Tübingen, and the abstract from his paper, given below, cannot, we think, fail to interest our readers.

The paper is based upon cases treated in the three years from February, 1911, to February, 1914. In that period there were 102 operations for gastric ulcer, of which 55 were gastro-enterostomies, 35 resections, and 12 operations for acute perforations. It is mainly with the group of resections that the writer deals.

*Ulcers of the Smaller Curvature of the Stomach.*—In the German Surgical Congress of 1912 Albert Kocher expressed the view that all forms of gastric ulcer, whether at the pylorus or elsewhere, were favourably influenced by gastro-enterostomy.

[1] *Deutsche Ztschr. f. Chir.*, 1914, cxxix. 464.

Perthes has not found this to be the case. He agrees with all or most surgeons that gastro-enterostomy leads to the healing of pyloric ulcers, but has found that ulcers of the small curvature of the stomach are often quite uninfluenced by the operation. It is partly by radiography, partly by secondary operations that this fact has been established. Radiography clearly showed in three cases quoted that callous ulcers of the lesser curvature of the stomach had persisted and enlarged in spite of successful gastro-enterostomies performed a long time previously. The first case showed that the ulcer had penetrated deeper, forming a typical accessory-cavity to the stomach (*Nebenhöhle*), although the hyperacidity existing before gastro-enterostomy had disappeared and bile was present in the stomach contents. The gastro-enterostomy opening had in this case been acting well for two years. In the second case the gastro-enterostomy had acted well for five years, but the ulcer had become much deeper.

The three cases showed further that it was the spastic contraction of the stomach at or near the place of ulceration which caused the advance of the disease. Other writers (Aschoff, Bergmann) had previously pointed out how ulcers set up reflex spasmodic contractions of the stomach, on the greater curvature and at the pylorus, which in turn caused local or general retention of stomach-contents adversely affecting the ulcers. Perthes found in fourteen cases of penetrating ulcer of the small curvature submitted to radiography that in six the spasmodic contraction of the stomach was in the ulcerated segment, but in the remaining eight the ring of contraction was immediately distal to the ulcer. Many times the spasmodic stenosis was tubular and of extraordinary narrowness. It appears that in many cases there is a vicious circle, the ulcer sets up spasm, the spasm causes advance and chronicity of the ulceration. Gastro-enterostomy does not prevent the occurrence of these spastic hour-glass stomach contractions, but the contractions prevent the anastomotic opening favourably influencing the ulcers. On the other hand, transverse resection of the portion of the stomach bearing the ulcer cured both ulcer and spasm.

Not only may ulcers of the small curvature set up spasmodic contractions near the ulcer, but, as observed by the writer in numerous cases, they caused spasm of the pylorus with distention of a second distal segment of the stomach. All forms of spasm, cease after transverse resection and the stomach empties itself normally or quicker than normally. Perthes concludes that the proper proceeding for callous penetrating ulcer of the smaller curvature is transverse resection of the stomach, and he also regards such resection as the operation of choice in ulcers of the small curvature which are not callous.

*Ulcers of the Pylorus.*—The indications for the treatment of pyloric ulcers by resection are not so definite. For simple ulcers, active or cicatrising, it is unquestionable that gastro-enterostomy is the operation of choice, with or without pyloric exclusion. Callous ulcers too are frequently cured by the operation. But in callous ulcers the relation of ulceration to the development of carcinoma has a bearing in the choice of operative methods of treatment. In this connection Perthes discusses separately two aspects of the problem, the possibility of mistaking the diagnosis and the possibility of the secondary development of carcinoma in the ulcer.

It is well known that neither previous clinical investigation, nor exploratory laparotomy, nor naked-eye examination of specimens, will always enable the distinction to be made between a callous ulcer and a carcinoma. Even the microscopical distinction in the hands of skilled pathologists presents many difficulties. As to the frequency of malignant change in ulcers of the stomach opinion is much divided. Investigations may be made to establish the percentage of gastric ulcers which show malignant change, or cases of carcinoma may be investigated to discover the percentage of them which appear to have begun as simple ulcers. Perthes considers that the duration of symptoms in cases of carcinoma affords light. In 119 cases out of 156 the symptoms of stomach trouble had not lasted more than two years, and this seems to show that in the vast majority of cases the carcinoma was primary. In fifteen cases there had been symptoms for more than ten years, suggesting that the trouble began in them with simple ulceration. Again, in four cases hæmatemesis had occurred at intervals for more than three years, suggesting primary simple ulceration. But such a history is rare, and again points to the conclusion that it is rare for carcinoma to begin as a simple ulcer. Another fact, pointing the same way, is that very few cases are recorded by operators in which a patient has died from gastric carcinoma after gastro-enterostomy performed for supposed ulcer. But when these statistics concern callous ulcers only, the subsequent death-rate from cancer is greater.

Perthes, in view of the difficulty of diagnosis, and the undoubted danger there is of malignant transformation, adopts the practice of resecting callous ulcers of the pylorus when the patient's condition is favourable and the resection presents no special technical difficulties. The suggestion is made that as the secondary carcinomatous tissue can be more easily found in the glands than the stomach ulcers themselves, it would be well if during operation a pathologist made a section of a gland by the freezing method and reported to the surgeon, a procedure which could be carried out in ten minutes.

*Technique of Stomach Resection.*—The most suitable and successful cases of resection are those performed for penetrating ulcer of the small curvature. The operation is easier if a transverse abdominal incision be added to the vertical one. It is made two fingers breadth above the umbilicus, and carried to the thoracic margin. Before dividing the rectus muscle and its sheath the writer sutures the muscle and sheath with catgut sutures, passed in a transverse row, three sutures above and three below the line of the intended division. These retaining sutures prevent retraction of the rectus muscle and much facilitate closure of the wound later, for the muscle can be neglected and the two fascial layers, anterior and posterior, alone sutured. If the ulcer cannot be separated from adherent parts without opening the stomach cavity, then the stomach contents, if any, are sucked out through a tube.

For the resection itself clamps are applied, slightly modified Lane clamps, and the suturing is carried out much as in gastro-enterostomy. When one segment of the stomach has a calibre larger than the other, this aperture is narrowed to the size of the smaller one by means of a "tobacco-pouch suture," which is tightened up to the necessary degree. This gathering suture does not interfere with the sutures applied later to join the two stomach segments.

If the pylorus has to be resected for cicatricial stenosis or cancer, then the junction of the stomach to the intestine may be well carried out by Reichel's modification of " Billroth's method ii." This modification consists in uniting the side of the highest part of the jejunum to the severed end of the stomach. In carrying this out the size of the stomach opening may well be diminished as described above.

*Results of the Operation.*—Simple transverse resection was done for seventeen cases of ulcer, and there was no death. The pylorus was resected seventeen times with two deaths. No abdominal hernia followed the operations. The results were favourable in all the cases, fifteen out of seventeen of the former class, which could be followed up. But of the fifteen cases traced eight had a more or less constant feeling of hunger. Many improved in this respect with time, but others had to take some food every two hours to feel comfortable.

X-ray examinations have thrown some light on this phenomenon of hunger. Two types of results were demonstrated. In both types the stomach was naturally smaller than before operation. In this first type the stomach appears as a uniformly wide sack or tube united at its lowest point to the pylorus. In the second type, with on the whole a larger stomach, there is present a considerable bulging on the lower curvature, and the pylorus lay not at the lowest part of the stomach, but higher up near the small curvature. In the second type more of the great curvature had been left at the operation.

In cases of the first type the food passed quickly through the open pylorus into the duodenum. In cases of the second type there were traces of the barium meal still in the stomach after five or six hours. The cases in which hunger was an after-symptom were those with the first type of stomach. This shows that a good portion of the greater curvature should be left at the operation. All the patients increased in weight and improved in general condition, and there was no trouble with over- or under-action of the bowels.

**The function of the gastro-enterostomy opening in cases of Permeable Pylorus.**—Henri Hartmann, of Paris, brings forward interesting points in discussing the above problem. The problem is a double one, and stated in the form of two questions :—

1. *Does the anastomotic opening become obliterated if the pylorus remains patulous ?* Kelling suggested that the opening might undergo anatomical obliteration if chyme passed by the pylorus and not by the anastomotic opening, and this opinion has been widely accepted. Hartmann does not support it, and writes that " when we refer to the cases of anatomical obliteration we see that these obliterations are in no way connected with the permeability of the pylorus."

In forty-five cases of such obliteration gathered from literature the writer only found four with permeable pylorus. In seven cases the obliteration was due to the cicatrisation of a peptic ulcer at the anastomotic opening. In twenty-three cases of obliteration buttons were used, in four cases sutures only, and in three Y-shaped gastro-enterostomy was performed.

2. *Are the gastro-intestinal anastomoses functionally useless in cases of permeable pylorus ?* In answer to this question the writer quotes Guibé,[1] who wrote in 1908, " All the experiments in animals and observations on men seem to agree sufficiently to prove that as long as the pylorus remains permeable, the stomach has an almost invincible tendency to drive out its contents through this orifice without being inclined to utilise the artificial mouth. Nothing whatever passes through the new opening ; on the contrary, everything passes through the pylorus."

Hartmann next quotes many recorded observations in which X-ray examinations were made, and in which it was clear that with permeable pylorus the gastric contents passed either wholly through the anastomotic opening or through that and the pylorus. Then he records experiments carried out on dogs, which showed that the evacuation is made principally by the anastomotic opening if that is situated in the pyloric antrum, but through the pylorus if the anastomotic opening is situated

[1] *J. de Chir.*, 1908, i. 1.

in the fundus of the stomach. The explanation lies in the pressure exerted on the chyme by the stomach contractions. which are much stronger in the pyloric segment of the stomach,

Radiological examinations in man have confirmed the above views. The examinations must, however, be skiascopic. Hartmann has almost always made the anastomosis on the lowest part of the pyloric antrum. Examination of nineteen patients at intervals of one to eleven years after operation showed that once everything passed through the pylorus, eleven times everything passed through the anastomosis, seven times the passage was through both openings.

J. Lacy Firth.

